# Intranasal Administration of Interferon Beta Attenuates Neuronal Apoptosis via the JAK1/STAT3/BCL-2 Pathway in a Rat Model of Neonatal Hypoxic-Ischemic Encephalopathy

**DOI:** 10.1177/1759091416670492

**Published:** 2016-09-28

**Authors:** Brandon J. Dixon, Di Chen, Yang Zhang, Jerry Flores, Jay Malaguit, Derek Nowrangi, John H. Zhang, Jiping Tang

**Affiliations:** 1 Department of Physiology and Pharmacology, Loma Linda University School of Medicine, CA, USA; 2 Department of Neurosurgery, Loma Linda University School of Medicine, CA, USA

**Keywords:** anti-apoptosis, infarct volume, interferon beta, intranasal administration, neonatal stroke, neuronal degeneration

## Abstract

Neonatal hypoxic-ischemic encephalopathy (HIE) is an injury that often leads to detrimental neurological deficits. Currently, there are no established therapies for HIE and it is critical to develop treatments that provide protection after HIE. The objective of this study was to investigate the ability of interferon beta (IFNβ) to provide neuroprotection and reduce apoptosis after HIE. Postnatal Day 10 rat pups were subjected to unilateral carotid artery ligation followed by 2.5 hr of exposure to hypoxia (8% O_2_). Intranasal administration of human recombinant IFNβ occurred 2 hr after HIE and infarct volume, body weight, neurobehavioral tests, histology, immunohistochemistry, brain water content, blood–brain barrier permeability, enzyme-linked immunosorbent assay, and Western blot were all used to evaluate various parameters. The results showed that both IFNβ and the Type 1 interferon receptor expression decreases after HIE. Intranasal administration of human recombinant IFNβ was able to be detected in the central nervous system and was able to reduce brain infarction volumes and improve neurological behavior tests 24 hr after HIE. Western blot analysis also revealed that human recombinant IFNβ treatment stimulated Stat3 and Bcl-2 expression leading to a decrease in cleaved caspase-3 expression after HIE. Positive Fluoro-Jade C staining also demonstrated that IFNβ treatment was able to decrease neuronal apoptosis. Furthermore, the beneficial effects of IFNβ treatment were reversed when a Stat3 inhibitor was applied. Also an intraperitoneal administration of human recombinant IFNβ into the systemic compartment was unable to confer the same protective effects as intranasal IFNβ treatment.

## Introduction

Neonatal hypoxic-ischemic encephalopathy (HIE) is a devastating disease that primarily causes neuronal and white matter injury. HIE has tremendous detrimental effects on the developing brain and is among the leading causes of death among infants, as well as the major underlying cause of seizures in term infants (Volpe, 2001; Doycheva et al., 2013; Shetty, 2015). Although there have been major advances in modern technology and an increased understanding of fetal and neonatal pathologies, HIE is still a serious condition that is unresolved and causes significant mortality and long-term morbidity (Badr Zahr and Purdy, 2006; Gill and Perez-Polo, 2008; Northington et al., 2011; Shankaran, 2012). These adverse events in the developing brain often lead to long lasting detrimental neurological defects later on in life such as mental retardation, epilepsy, cerebral palsy, learning disabilities, and other neurophysiological handicaps (Fathali et al., 2010; Li et al., 2016). Currently, there are no specific treatments to repair the damage caused by HIE (Zhu et al., 2013; Shaikh et al., 2015). Thus, it is critically important to develop safe and effective therapies (Ramanantsoa et al., 2013; Caltagirone et al., 2016).

Neonatal HIE can also be characterized as an injury that occurs in the immature brain, resulting in delayed cell death via excitotoxicity, inflammation, and oxidative stress (Bain et al., 2013; Dixon et al., 2015). Previous studies have shown that the newborn brain is primed to respond to various insults with the activation of apoptotic cascades since cell death is a normal part of development in the central nervous system (CNS). Thus, there is a high expression of pro-apoptotic proteins in the developing brain (Northington et al., 2011). As a consequence, mitochondrial dysfunction occurs and ultimately signals pathways of apoptosis (Qi et al., 2015; Alhadidi et al., 2016). Specifically, the release of cytochrome c by the mitochondria leads to activation of caspase-9 followed by active caspase-3 between 6 and 48 hr after injury (Gill and Perez-Polo, 2008).

Clinically, interferon beta (IFNβ) is the primary treatment used to combat inflammation and flare-ups in multiple sclerosis (Johnston and So, 2012; Castrop et al., 2013). IFNβ is able to increase expression and concentration of anti-inflammatory cytokines, while also having the effect of decreasing the expression of pro-inflammatory agents (Kieseier, 2011). However, the neuroprotective properties and mechanisms of IFNβ have not yet been explored following HIE.

The administration of human recombinant IFNβ as a treatment is a novel approach in neonatal HIE since it is already a food and drug administration approved treatment for multiple sclerosis (English and Aloi, 2015). Intrastriatal injections of IFNβ have been shown to preserve the blood–brain barrier integrity, decrease infarct size, and block the infiltration of inflammatory cells in an adult rat model of middle cerebral artery occlusion (Veldhuis et al., 2003). These studies indicate that IFNβ may have some anti-apoptotic and anti-inflammatory properties in the CNS after significant damage like HIE.

IFNβ specifically acts through the Type 1 interferon receptor (IFNR) which has been shown to be expressed on endothelial cells and leukocytes (Ross et al., 2004; Johnston and So, 2012). It has been characterized that after IFNR activation, the Jak-Stat pathway is triggered for positive feedback of IFNβ and activation of several other pathways that are associated with anti-apoptosis (Heim, 1999; Schindler et al., 2007; Zula et al., 2011). As a result of IFNβ binding to the receptor, one product in neurons is Stat3 (Schindler et al., 2007; Dziennis and Alkayed, 2008; Ramgolam et al., 2011). It has been shown that Stat3 leads to the increased transcription of Bcl-2, which lowers the Bax/Bcl-2 ratio (Dziennis and Alkayed, 2008). Interestingly, p53 increases after HIE injury and induces the increase of Bax (Northington et al., 2011). This causes a high Bax/Bcl-2 ratio leading to Bax-mediated mitochondrial permeabilization, which is also known to increase after HIE (Wullner et al., 1998; Pan et al., 2012; Thornton and Hagberg, 2015). These events can lead to the activation and cleavage of caspases, specifically caspase-9 and caspase-3 (Wullner et al., 1998; Pan et al., 2012; Thornton and Hagberg, 2015). Thus, we were able to derive the hypothesis that intranasal delivery of human recombinant IFNβ will provide protection through its anti-apoptotic properties after HIE.

## Materials and Methods

All protocols and experiments in this study were approved by the Institutional Animal Care and Use Committee of Loma Linda University. All animals were handled in accordance with the National Institute of Health Guide for the Care and Use of Laboratory Animals.

### Neonatal HIE Animal Model

A modified Rice-Vannucci model was used as previously described in our past publications (Fathali et al., 2013; Drunalini Perera et al., 2014; Charles et al., 2015). Postnatal Day 10 Sprague-Dawley rat pups purchased from Harlan Laboratories (Livermore, CA) were anesthetized and underwent a unilateral right common carotid artery ligation using a 5-0 surgical silk suture. After a recovery period of 1 hr, the rat pups were placed in a submerged 37° hypoxia chamber (8% O_2_) for 2.5 hr. The flow rate of the gas 93.82 mL/min for the first 1.25 hr and was 77.30 mL/min for the remaining 1.25 hr. Sham animals also underwent anesthesia and exposure of the carotid artery; however, the artery was not ligated.

### Postsurgical Care

After the surgeries, the rat pups were returned to their mothers. The rat pups were monitored closely for bleeding, swelling, pain, and distress. The monitoring occurred every 20 min for the first 2 hr, at 6 hr, and then once daily until sacrifice.

### Drug Administration

Intranasal administration of IFNβ (0.03 µg/kg, 0.3 µg/kg, and 1.0 µg/kg) was applied 2 hr after HIE in the treatment group. The best dose (0.3 µg/kg) was chosen and administered every 24 hr until the 72-hr time point and then was used throughout the duration of the study. A specific Stat3 inhibitor, WP1066 (0.5 µg/kg), was applied via intraperitoneal injection 1 hr before HIE in combination with IFNβ treatment. In addition, one group of animals received 0.3 µg/kg IFNβ treatment via intraperitoneal injection. The vehicle used to dissolve the IFNβ tablets was deionized water which was administered to the sham and vehicle groups. Human recombinant IFNβ (Avonex) was purchased from the Loma Linda University Medical Center Pharmacy (Loma Linda, CA), and WP1066 was obtained from Abcam (Cambridge, MA).

### Intranasal Administration

Intranasal administration was performed as previously described (Topkoru et al., 2013; Zhang et al., 2015). Rat pups were anesthetized with isoflurane and placed on their backs in an anesthesia chamber. Underneath the anesthesia chamber, a heating pad was used to maintain the temperature. A rolled pad was placed under the necks of the rat pups to keep the heads stable. After the treatment, the rat pups were monitored until they were fully conscious and exhibited normal breathing.

### 2, 3, 5-Triphenyltetrazolium Chloride Monohydrate Staining

At the 24- and 72-hr time points, the rat pups were anesthetized and perfused transcardially with phosphate-buffered saline (PBS). The brains were removed, sectioned into 2 mm slices, and immersed into a 2% 2, 3, 5-Triphenyltetrazolium Chloride Monohydrate (TTC) solution at 37℃ for 5 min as routinely performed (Chen, Burris, et al., 2011; Dock et al., 2015). The samples were then rinsed with PBS and stored in a 10% formaldehyde solution or underwent preparation for Western blot to be utilized in the protein expression evaluation groups as previously described (Kramer et al., 2010). The infarct volume was traced and analyzed with Image J Software.

### Western Blot

The Western blot experiments were executed as routinely performed (Zheng et al., 2015; Sherchan et al., 2016). Rat pups were euthanized under isoflurane followed by the preparation of brain tissue samples. Tissues samples were created by collecting and snap freezing the brain hemispheres with liquid nitrogen followed by storage at −80℃. Prior to the Western blot sample preparation, these samples were stained with TTC since they are shared with the infarct evaluation groups. Cytosolic fractionation extracts were obtained from the brain samples. Denatured protein extract (50 µg) was electrophoresed and transferred to a nitrocellulose membrane and probed with antibodies. The following primary antibodies were used: Interferon beta-receptor (1:500; Santa Cruz Biotechnology), IFNβ (1:500; Santa Cruz Biotechnology), Stat-3 (1:500; Abcam), Bcl-2 (1:1000; Cell Signaling), and cleaved caspase 3 (1:500; Cell Signaling). The membranes were then incubated with the appropriate secondary antibodies (Santa Cruz Biotechnology and Thermo Fisher). The optical densities of the bands were visualized using ECL Plus (GE Healthcare Life Sciences) or Li-Cor fluorescence technology and were analyzed with Image J Software (National Institute of Health).

### Enzyme-Linked Immunosorbent Assay (ELISA)

Rat pups were euthanized under isoflurane anesthesia, and the brain hemispheres were collected, snap frozen in liquid nitrogen, and stored at −80℃. Human recombinant IFNβ from the cytosolic fractionation extracts was then detected using a commercial ELISA kit (Thermo Scientific).

### Histology and Immunohistochemistry

Immunohistochemistry was conducted as routinely executed (Chen, Zhang, et al., 2015; Huang et al., 2015). The rat pups were perfused under anesthesia with PBS followed by 4% formaldehyde. The brains were removed for postfixation in formalin. The paraffin-embedded brains were then sectioned into 10 µm slices via cryostat. The brain slices were then evaluated with immunohistochemistry and a Fluoro-Jade C staining kit (Biosensis). The specific cellular marker antibody used was NeuN (1:100; Abcam). The brain slices were then incubated with the appropriate fluorescent secondary antibodies (Jackson ImmunoResearch). The brain slices were then visualized with a fluorescent microscope (Olympus BX51 and Keyence BZ-9000) under 20×, 40×, and 60 × magnifications with an aperture of 0.75, 0.95, and 0.95, respectively.

### Short-Term Neurobehavioral Tests

Both the righting reflex test and the geotactic reflex test were performed as previously described (Zhang et al., 2013; Yuan et al., 2014). Both short-term neurobehavioral tests were conducted at the 24-hr time point and continued to the 72-hr time point after HIE. The animals in these groups were also shared with the TTC and Western blot groups.

#### Righting reflex test

The righting reflex test was performed by placing the rat pups in a supine position on a table surface and recording the amount of time for the animal to reach a prone position, where all four paws are against the surface of the table.

#### Geotactic reflex test

The geotactic reflex test was performed by placing the rat pups in a downward orientation on a 45° incline. The amount of time for the rat pup to recognize its position on an incline, make a full 180° turn, and face the top of the incline was recorded.

### Brain Water Content

Brain water content was performed as previously conducted (Chen et al., 2009; Li, Tao, et al., 2015). The rat pups were placed under isoflurane anesthesia and were decapitated 24 hr after injury, and the brain hemispheres were quickly separated and removed. All tissue samples were weighed using an analytical microbalance in order to obtain the wet weight. The samples were then dried at 100℃ for 24 hr before determining the dry weight. Brain water content (%) was calculated as (wet weight − dry weight)/wet weight × 100.

### Evan's Blue Dye Extravasation Assay

Evan's Blue dye extravasation was executed as previously described (Li, McBride, et al., 2015; Merali et al., 2015; Dixon et al., 2016; Zhang et al., 2016). Evan's Blue dye was administered via intraperitoneal injection and allowed to circulate 6 hr before the 24-hr time point to measure blood–brain barrier permeability. At the 24-hr time point, the rat pups were sacrificed under anesthesia and perfused with PBS. The brain hemispheres were then collected and homogenized in a 3:1 ratio of 50% trichloroacetic acid to brain tissue mass. The supernatant was then analyzed at 620 nm by spectrophotometry.

### Statistical Analysis

*T*-test comparisons, one-way analysis of variance, Newman-Keuls multiple comparison test, and Tukey's post hoc analysis were performed in GraphPad Prism.

## Results

### Expression of IFNβ and IFNR in the CNS After HIE

**Figure 1.**
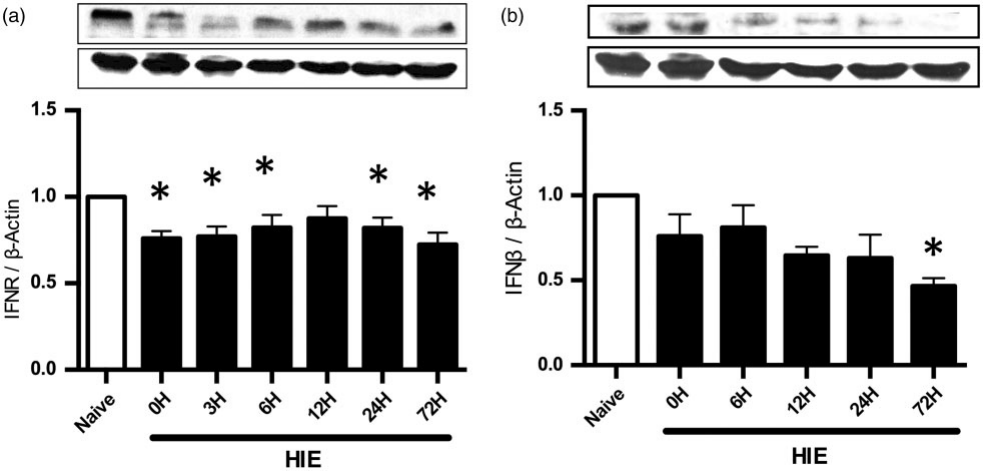
Endogenous Type 1 interferon receptor and interferon beta expression in the injured hemisphere of the neonatal central nervous system. (a) Western blot analysis of the injured brain hemisphere illustrates that IFNR expression decreased after 24 hr in the central nervous system. (b) Western blot analysis of the injured brain hemisphere demonstrates that IFNβ protein expression decreases at 72 hr after injury. Significance determined based on comparison to the naïve control group (*p < .05) as calculated by student's *T* test. HIE = hypoxic-ischemic encephalopathy; IFNβ = interferon beta.

IFNR expression decreases shortly after HIE insult and is also significantly different from the naïve control group after 24 hr and 72 hr (Figure 1(a)). IFNβ significantly decreases after HIE at the 72-hr time point when compared with the naïve control group (Figure 1(b)). There are no significant differences in protein expression 0 to 72 hr after HIE for both IFNβ and IFNR (IFNβ: *N* = 3 rats per group; IFNR: *N* = 4 rats per group). Significance determined based on comparison to the naïve control group (*p < .05) as calculated by student's *T* test.

### Exogenous IFNβ Reaches the CNS After Intranasal Administration

**Figure 2.**
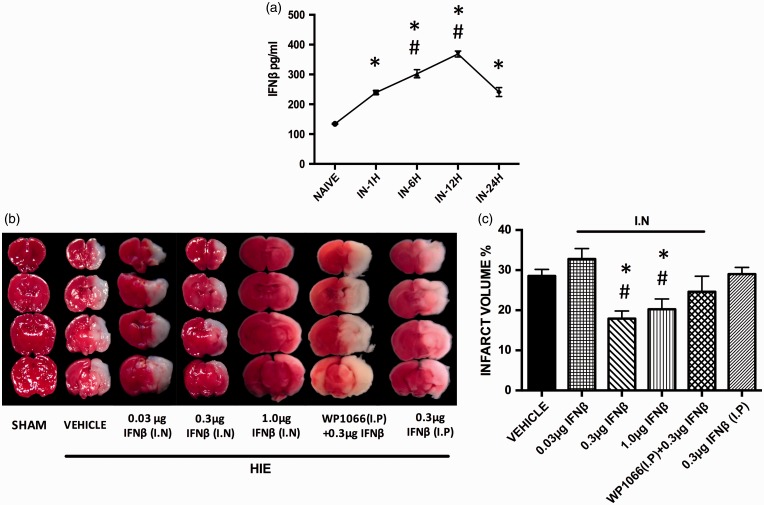
Intranasal administration of human recombinant interferon beta after 24 hr in the central nervous system. (a) A time course of exogenous IFNβ in the injured right hemisphere of the central nervous system after intranasal administration using an ELISA assay. Significance determined based on comparisons to the naïve control group and the IN-1H group (*p < .05 vs. Naive; ^#^*p* < .05 vs. IN-1H) as calculated by the Newman-Keuls multiple comparison test. (b) Illustrates the effects of IFNβ intranasal treatment with TTC staining of 2 mm brain slices 24 hr after neonatal hypoxic-ischemic encephalopathy. (c) Displays that intranasal administration 0.3 µg and 1.0 µg IFNβ decreases infarct size 24 hr after injury. Intraperitoneal administration of IFNβ did not reduce brain infarct volume. The effect of intranasal IFNβ was reversed when coadministered with a Stat3 inhibitor (WP1066). Significance determined based on comparisons to the HIE+Vehicle and HIE+0.03 µg IFNβ groups (*p < .05 vs. HIE+Vehicle; ^#^*p* < .05 vs. HIE+0.03 µg IFNβ) as calculated by Tukey's test. HIE = hypoxic-ischemic encephalopathy; IFNβ = interferon beta.

A time course of a single dose of human recombinant IFNβ intranasal administration was performed. Intranasal IFNβ 0.3 µg/kg (*N* = 6 rats per group) significantly increased 1 hr after the initial administration, and peak concentrations were observed at the 12-hr time point. IFNβ began to decline after 24 hr; however, it was still significantly higher than the control group (Figure 2(a)). Significance determined based on comparisons to the naïve control group and the IN-1H group (*p < .05 vs. Naive; ^#^*p* < .05 vs. IN-1H) as calculated by the Newman-Keuls multiple comparison test.

### Intranasal Administration of IFNβ Decreased Infarct Volume 24 hr After HIE

Intranasal IFNβ medium, HIE + 0.3 µg IFNβ (*N* = 10 rats per group) and high dose HIE + 1.0 µg IFNβ (*N* = 6 rats per group) treatments were administered at 2 hr following HIE. Both doses were able to decrease infarct volumes after 24 hr when compared with the other control groups (*p < .05 vs. HIE + Vehicle; ^#^*p* < .05 vs. HIE + 0.03 µg IFNβ; Figure 2(b) and (c)). The low dose group, HIE + 0.03 µg IFNβ (*N* = 6 rats per group), had no effect on infarct volume when compared with the HIE + Vehicle group after 24 hr. Significance determined based on comparisons to the HIE + Vehicle and HIE + 0.03 µg IFNβ groups (*p < .05 vs. HIE + Vehicle; ^#^*p* < .05 vs. HIE + 0.03 µg IFNβ) as calculated by Tukey's test.

### Intraperitoneal Administration of IFNβ as well as cotreatment of a Stat3 Inhibitor and Intranasal IFNβ Fails to Reduce Infarct Volumes 24 hr After HIE

Intraperitoneal injection of IFNβ (0.3 µg) was ineffective in reducing infarct volume 24 hr after injury (*N* = 4 rats per group). Also the protective effects of intranasal administration of IFNβ were diminished when WP1066 (0.5 µg/kg), a Stat3 inhibitor, was administered in combination with IFNβ treatment at 24 hr post injury. The HIE + WP1066 + 0.3 µg IFNβ group (*N* = 5 rats per group) was not significantly different from the HIE + Vehicle group (Figure 2(b) and (c)).

### Intranasal Administration of IFNβ Has no Effect on Brain Water Content and Blood–Brain Permeability at 24 hr After HIE

**Figure 3.**
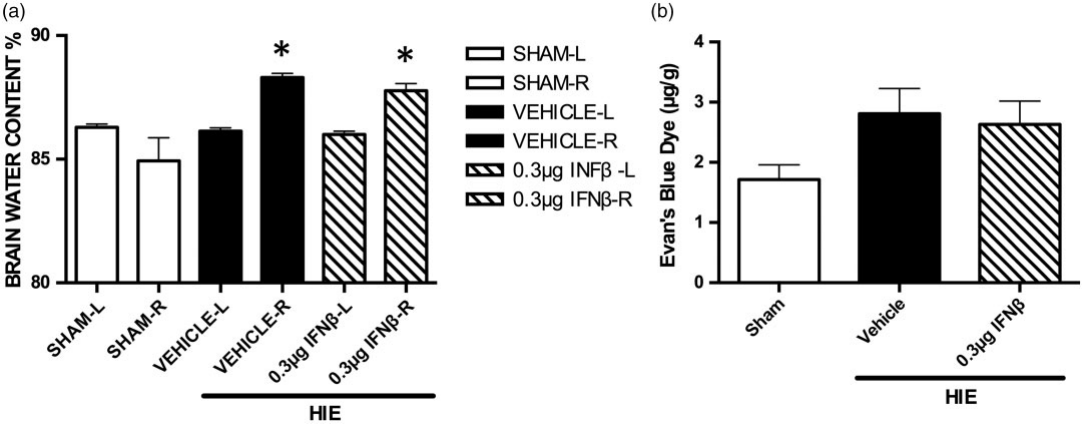
The effects of intranasal administration of human recombinant interferon beta on brain water content and blood barrier permeability after 24 hr after hypoxic-ischemic encephalopathy. (a) Displays the brain water content of each hemisphere of the brain 24 hr after hypoxic-ischemic encephalopathy, indicating brain edema in the injured right hemisphere. Significance determined based on comparisons to the Sham group (*p < .05 vs. Sham) as calculated by Tukey's test. (b) The amount of Evan's Blue extravasation in the hemispheres of the central nervous system after 24 hr after hypoxic-ischemic encephalopathy. No significance was determined as calculated by Tukey's test. HIE = hypoxic-ischemic encephalopathy; IFNβ = interferon beta.

Figure 3(a) shows the trend that IFNβ treatment can reduce brain water content at the 24-hr time point (*N* = 7 per group). Brain water content in the injured right hemisphere was significantly increased in both the HIE + Vehicle and HIE + IFNβ groups when compared with sham controls. Significance determined based on comparisons to the Sham group (*p < .05 vs. Sham) as calculated by Tukey's test. This indicates that at 24 hr, IFNβ treatment may not play a strong role in reducing brain edema but plays a more anti-apoptotic role. Similarly, Figure 3(b) also shows the trend that IFNβ treatment may decrease blood–brain barrier permeability (*N* = 7 per group). No significance was determined as calculated by Tukey's test.

### Intranasal Administration of IFNβ Decreases Infarct Volume and Improves Short-Term Neurobehavioral 72 hr After HIE

**Figure 4.**
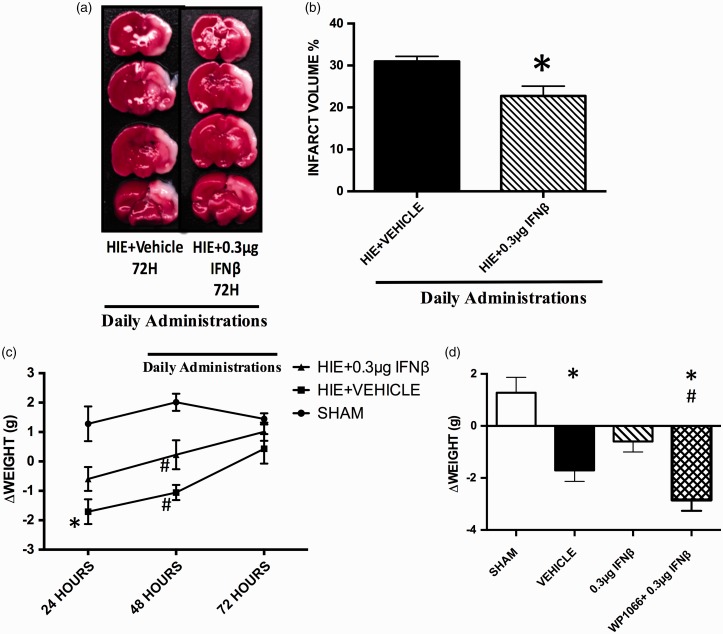
The effects of daily interferon beta administration after 72 hr. (a) Illustrates TTC staining of 2 mm brain slices 72 hr after hypoxic-ischemic encephalopathy. (b) Displays that intranasal 0.3 µg IFNβ decreases infarct size 72 hr after injury. Significance determined based on comparisons to the HIE+Vehicle group (*p < .05 vs. HIE+Vehicle) as calculated by student's *T* test. (c) A time course of changes in weight after hypoxic-ischemic encephalopathy. Significance determined based on comparisons to the Sham 24-hr group and the Sham 48-hr group (*p < .05 vs. Sham- 24 hr; ^#^*p* < .05 vs. Sham- 48 hr) as calculated by Tukey's test. (d) The effects of IFNβ treatment on weight at 24 hr. Significance determined based on comparisons to the Sham and 0.3 µg IFNβ (*p < .05 vs. Sham; ^#^*p* < .05 vs. 0.3 µg IFNβ) as calculated by Tukey's test. HIE = hypoxic-ischemic encephalopathy; IFNβ = interferon beta.

Daily intranasal administration of IFNβ 0.3 µg treatment (*N* = 7 rats per group) also significantly reduced infarct volume when compared with the HIE + Vehicle group (*N* = 7 rats per group) at the 72-hr time point as well (Figure 4(a) and (b)). Significance determined based on comparisons to the HIE + Vehicle group (*p < .05 vs. HIE + Vehicle) as calculated by student's *T* test. The daily administration of 0.3 µg IFNβ treatment was able to improve weight loss after injury (Figure 4(c) and (d)). At the 24-hr time point, the HIE + Vehicle group lost a significant amount of weight when compared with the Sham group. The HIE + 0.3 µg IFNβ was not significantly different from the Sham group (*p < .05 vs. Sham; Figure 4(c) and (d)). At the 48-hr time period, both the IFNβ treatment and Vehicle were significantly different from the Sham group (^#^*p* < .05 vs. Sham). Significance determined based on comparisons to the Sham 24-hr group and the Sham 48-hr group (*p < .05 vs. Sham- 24 hr; ^#^*p* < .05 vs. Sham- 48 hr) as calculated by Tukey's test. Also at the 24-hr time point, the decrease in weight loss effect of IFNβ treatment was reversed in the WP1066 + 0.3 µg IFNβ (*N* = 5 per group). Significance determined based on comparisons to the Sham and 0.3 µg IFNβ (*p < .05 vs. Sham; ^#^*p* < .05 vs. 0.3 µg IFNβ) as calculated by Tukey's test.

**Figure 5.**
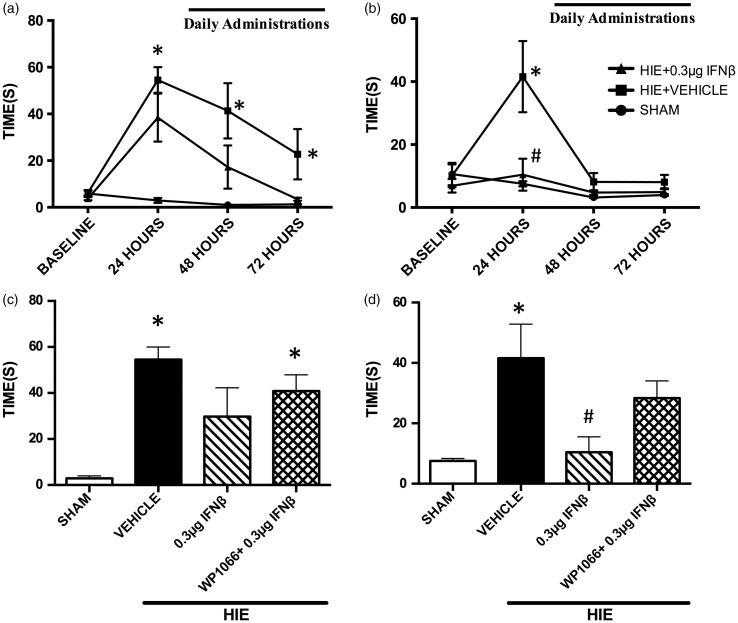
Short-term neurobehavioral tests after hypoxic-ischemic encephalopathy. (a) A time course of righting reflex behavior times after hypoxic-ischemic encephalopathy. (b) A time course of geotactic neurobehavior times after hypoxic-ischemic encephalopathy after 72 hr. (c) Intranasal treatment of 0.3 µg IFNβ improved righting reflex times at the 24-hr time point. Significance determined based on comparisons to the Sham group (*p < .05 vs. Sham) as calculated by Tukey's test. (d) Intranasal treatment of 0.3 µg IFNβ improved geotactic reflex times at the 24-hr time point. Significance determined based on comparisons to the Sham and Vehicle groups (*p < .05 vs. Sham; ^#^*p* < .05 vs. Vehicle) as calculated by Tukey's test. HIE = hypoxic-ischemic encephalopathy.

Figure 5 displays data acquired from short-term neurobehavioral tests up to the 72-hr time point. The righting reflex test, a motor functioning test, shows that the HIE + Vehicle group (*N* = 5 rats per group) required a significantly longer time to correct their positioning when compared with the Sham groups (*N* = 7 rats per group) indicating physical dysfunction at 24 hr. The HIE + Vehicle was also significantly increased at the 72-hr time point as well. Interferon treatment was able to decrease righting reflex time and was not significantly different from the Sham group. The protective effect of IFNβ was diminished in the HIE + WP1066 + 0.3 µg IFNβ group (*N* = 5 rats per group; Figure 5(c)). Significance determined based on comparisons to the Sham group (*p < .05 vs. Sham) as calculated by Tukey's test. The geotactic reflex test illustrates similar results to the righting reflex test (Figure 5(b)). Animals that received IFNβ intranasal treatments were able to recognize that they were on an inclined surface and turned 180° faster, when compared with the vehicle group. Furthermore, the effects of IFNβ were reversed, when WP1066 was used in combination with IFNβ treatment as well (*N* = 5 rats per group; *p < .05 vs. Sham; ^#^*p* < .05 vs. Vehicle; Figure 5(d)). Significance determined based on compare sons to the Sham and Vehicle groups (*p < .05 vs. Sham; ^#^*p* < .05 vs. Vehicle) as calculated by Tukey's test.

### Intranasal Administration of IFNβ Increases Anti-Apoptotic Proteins and Decreases Fluoro-Jade Positive Neurons

**Figure 6.**
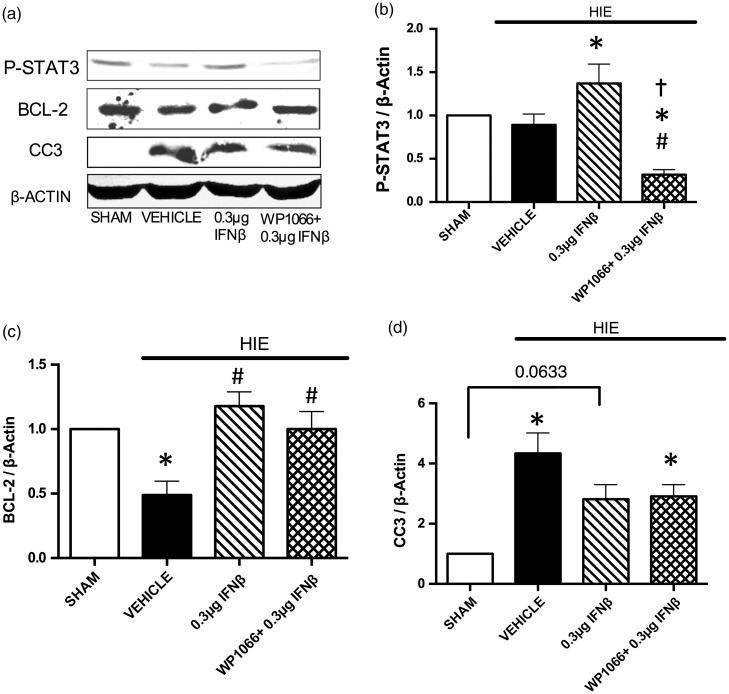
Expression of anti-apoptotic proteins involved with interferon beta treatment 24 hr after hypoxic-ischemic encephalopathy. (a) Representative Western blot images of P-STAT3, Bcl-2, and cleaved caspase-3 (CC3) expression in the injured right hemisphere of the brain 24 hr after hypoxic-ischemic encephalopathy. (b) Western blot analysis of P-STAT3 expression in the injured right brain hemisphere 24 hr after hypoxic-ischemic encephalopathy. Significance determined based on comparisons to the HIE+Vehicle, HIE+0.3 µg IFNβ, and Sham groups (*p < .05 vs. HIE+Vehicle; ^#^*p* < .05 vs. HIE+0.3 µg IFNβ; (*p* < .05 vs. Sham) as calculated by Tukey's test. (c) Western blot analysis of BCL-2 expression in the injured right brain hemisphere 24 hr after injury. Significance determined based on comparisons to the Sham and HIE+Vehicle groups (*p < .05 vs. Sham; ^#^*p* < .05 vs. HIE+Vehicle) as calculated by Tukey's test. (d) Western blot analysis of cleaved caspase-3 expression in the injured right brain hemisphere at 24 hr after injury. Significance determined based on comparisons to the Sham group (*p < .05 vs. Sham) as calculated by Tukey's test. HIE = hypoxic-ischemic encephalopathy.

**Figure 7.**
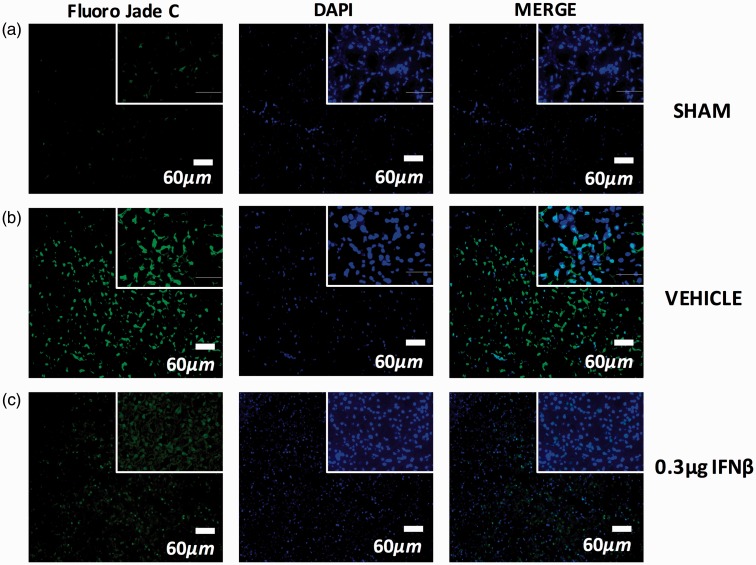
Illustrates positive Fluoro-Jade C expression in the central nervous system after 24 hr after injury. (a) Shows positive staining in the hippocampal region of the brain in the sham group at the 24-hr time point after utilizing immunohistochemistry and a Fluoro-Jade C kit. (b) Illustrates positive Fluoro-Jade C staining in the hippocampal region of the brain in the vehicle group after 24 hr using immunohistochemistry and a Fluoro-Jade C kit. (c) Immunohistochemistry and Fluoro-Jade C staining kit revealed positive Fluoro-Jade C staining in the hippocampal region of the brain after intranasal administration of 0.3 µg IFNβ. IFNβ = interferon beta.

Intranasal IFNβ significantly increased expression of anti-apoptotic proteins and decreased neuronal cell death (Figures 6 and 7). P-STAT3 expression was increased in the HIE + 0.3 µg IFNβ group when compared with the HIE + Vehicle group 24 hr post injury. Also P-STAT3 expression was significantly decreased after inhibition with WP1066 (*N* = 5 per group; Figure 6(b)). Significance determined based on comparisons to the HIE + Vehicle, HIE + 0.3 µg IFNβ, and Sham groups (*p < .05 vs. HIE + Vehicle; ^#^*p* < .05 vs. HIE + 0.3 µg IFNβ; (*p* < .05 vs. Sham) as calculated by Tukey's test. Bcl-2 expression was significantly decreased after HIE. Intranasal IFNβ treatment and IFNβ treatment in combination with STAT3 inhibition were able to increase Bcl-2 expression after HIE when compared with the vehicle (Figure 6(c)). Significance determined based on comparisons to the Sham and HIE + Vehicle groups (*p < .05 vs. Sham; ^#^*p* < .05 vs. HIE + Vehicle) as calculated by Tukey's test. Cleaved caspase-3 expression was significantly increased in the HIE + Vehicle and HIE + WP1066 + 0.3 µg IFNβ groups. Cleaved caspase-3 expression was lower in the HIE + 0.3 µg IFNβ after 24 hr and not significantly different from any of the other groups (*p* = .0633 vs. Sham; *N* = 5 rats per group for all groups; Figure 6(d)). Significance determined based on comparisons to the Sham group (*p < .05 vs. Sham) as calculated by Tukey's test.

After HIE, there was more positive Fluoro-Jade C expression in the hippocampal region in the HIE + Vehicle group. Intranasal IFNβ treatment decreased the expression of positive Fluoro-Jade C expression, the Sham group also had a decreased expression as well (Figure 7).

## Discussion

There are a few studies evaluating the effects of IFNβ treatment after ischemic stroke (Veldhuis et al., 2003; Marsh et al., 2009; Kuo et al., 2016). One of the earliest studies explored IFNβ treatment after middle cerebral artery occlusion in a rabbit model and found that IFNβ was neuroprotective and decreased infarct volumes (Liu et al., 2002). However, another study reported that IFNβ treatment failed following middle cerebral artery occlusion in a rat model. The authors of this study suggested that IFNβ treatment was unable to reach the affected tissues in the CNS since the blood–brain barrier was not sufficiently disrupted in their model (Maier et al., 2006).

Thus in our study, we chose intranasal administration of IFNβ treatment since it is clinically viable and noninvasive (Brown and Liu, 2014). Also intranasal delivery of IFNβ has previously been shown to effectively bypass the blood–brain barrier and directly target the CNS in a rat model of multiple sclerosis (Ross et al., 2004). To our knowledge, this is the first study to explore the effects of IFNβ treatment after neonatal HIE as well as the first to explore anti-apoptotic mechanisms involved with IFNβ in this model.

In the present study, we observed that overall expression of both the IFNR and IFNβ decreases in the CNS after neonatal HIE. We also found that intranasal IFNβ treatment was able to decrease infarction volumes after 24 hr and 72 hr, improve short-term neurobehavior at those time points, as well as improve postsurgery weight gain. Intranasal IFNβ treatment also had an anti-apoptotic effect of increasing Stat3 and Bcl-2 expression in addition to decreasing the expression of cleaved caspase-3 and neuronal degeneration. Moreover, pharmacological inhibition of Stat3 expression with the specific Stat3 inhibitor, WP1066, was able to reverse the protective effects of IFNβ that we observed. Pharmacological inhibition of Stat3 reversed protective effects of decreased infarction volumes and short-term behavior after HIE when applied in combination with intranasal IFNβ treatment.

The importance of Stat3 expression after stroke and neonatal HIE has been controversial. Our results show that IFNβ treatment increased Stat3 activation, the downstream product, after HIE. It has been previously suggested that treatments with the ability to activate Stat3 expression have been found to improve behavioral functions and decrease cell death after stroke (Raible et al., 2014). Our previous study demonstrating the effects of granulocyte-colony stimulating factor (G-CSF) in an experimental model of HIE observed that Stat3 was among some of the proteins contributing to anti-apoptosis in the study (Yata et al., 2007). Kinouchi et al. (2012) also found that activation of Stat3 in the peri-infarct region was associated with inhibition of apoptosis in a model of middle cerebral artery occlusion. Conversely, Hristova et al. observed that complete knockout of Stat3 in neurons and astrocytes after neonatal HIE in mice reduced brain damage and attenuated apoptosis by decreasing microglial activation, astroglial activation, and reactive astrogliosis. Interestingly, the same study also found that successful preinsult and postinsult pharmacological inhibition of Stat3 with WP1066 resulted in a weak response that did not reduce brain tissue loss (Hristova et al., 2016).

Similarly, we observed that pharmacological Stat3 inhibition in combination with IFNβ treatment also did not yield improvement to brain infarction volume. Also Stat3 inhibition in concert with IFNβ treatment did not improve short-term neurobehavior or increases in anti-apoptotic protein expression suggesting that the beneficial effects of IFNβ treatment are mediated through Stat3 expression.

Our previous publications have demonstrated that hypoxic ischemic injury in the CNS induces the expression of Bax leading to apoptotic cell death (Charles et al., 2012). We have also previously demonstrated that the Bcl-2 is significantly decreased 24 hr after HIE and that increases in its expression leads to decreases in cleaved caspase-3 and neuronal cell death (Charles et al., 2012; Li, Klebe, et al., 2015). Other studies have also shown that increases in Bcl-2 lead to a decrease in neuronal apoptosis as well (Hu et al., 2014; Chen, Xu, et al., 2015). Similarly, our results show that Bcl-2 is decreased 24 hr after HIE and that intranasal IFNβ is able to increase Bcl-2 expression after injury contributing to neuronal survival. We believe that this effect is mediated by an increase in Bcl-2 which was induced by Stat3 activation via IFNβ treatment. Bcl-2 expression was also elevated in the Stat3 inhibition group as well. Since we observed the whole cellular expression of Bcl-2 in the injured hemisphere it is possible that other cell types contributed to the elevation of Bcl-2 in response to Stat3 inhibition.

It has been previously shown that cleaved caspase-3 expression increases after HIE and peaks at the 24-hr time point contributing to neuronal apoptosis (Wang et al., 2001; Askalan et al., 2015). Increased expression of positive Fluoro-Jade C staining has been observed before after 24 hr after HIE (Aggarwal et al., 2014). Our studies also showed that cleaved caspase-3 expression increased after 24 hr in the HIE + Vehicle group and that there is increased positive staining of Fluoro-Jade C expression as well. In our studies, intranasal IFNβ treatment was able to decrease cleaved caspase-3 expression and neuronal apoptosis after 24 hr after injury.

Following HIE, sensorimotor functions and reflex maturation are severely affected as well (Sanches et al., 2012; Alexander et al., 2014). Our findings show that IFNβ treatment was able to improve sensorimotor deficits and reflex maturation delays since there was an improvement in the performance on the righting reflex and geotactic reflex short-term neurobehavioral tests after HIE. Body weights of the rat pups can also be used as an indicator of general health after HIE (Chen, Ma, et al., 2011), thus we recorded the weights of the rat pups each day after injury. Normally after HIE, there is a significant amount of loss in weight after injury as evidenced by the literature and our previous studies (Carty et al., 2008; Fathali et al., 2013). In the present study, our data show that IFNβ treatment was able to provide an improvement of general health since weight loss after injury was significantly reduced.

Our results also demonstrated that IFNβ treatment had no effect on brain edema and blood–brain barrier permeability at the 24-hr time point. This result helped us focus our attention upon the anti-apoptotic effects that IFNβ treatment had on neurons since we observed decreases in the infarction volume at the 24-hr time point. Our results also showed that there was no detectable blood–brain barrier disruption at 24 hr after neonatal HIE injury as the vehicle group is not significantly different from the sham control group. Previous studies of neonatal HIE from our laboratory and the literature confirm and report similar findings that detectable disruptions of the blood–brain barrier occur at 48 hr after neonatal stroke in the rat pup model (Fernandez-Lopez et al., 2012; Li, McBride, et al., 2015).

**Figure 8.**
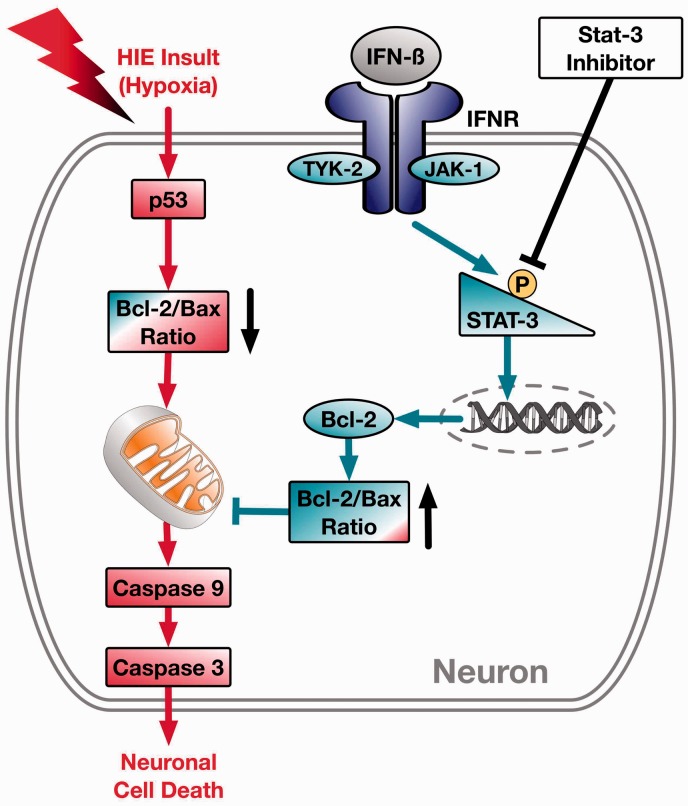
A visual schematic. In our experiments human recombinant interferon beta (IFN-β) administered through the intranasal route was able to bind and activate the Type 1 interferon receptor (IFNR). Activation of INFR leads to increases of P-Stat3 activation and transcription of Bcl-2, which increases the Bcl-2/Bax ratio. This eventually leads to the decreased expression of caspase 3 and ultimately decreases in neuronal cell death. IFNβ = interferon beta; IFNR = Type 1 interferon receptor.

In conclusion, we report that intranasal administration of human recombinant IFNβ treatment was able to attenuate neuronal apoptosis via the Jak1/Stat3/Bcl-2 pathway after neonatal HIE (Figure 8). We determined that intranasal human recombinant IFNβ treatment induces Stat3 activation and leads to increased expression of Bcl-2 which led to a decrease in cleaved caspase-3 expression and neuronal degeneration. IFNβ treatment has the potential to become a new therapeutic strategy after HIE, however more translational studies are still needed. Future studies should also explore potential anti-inflammatory mechanisms after IFNβ administration after HIE.

## Summary

Intranasal treatment with human recombinant IFNβ reduces brain injury and improves neurological functions after hypoxic-ischemic injury. These findings suggest that IFNβ could be a potential therapy for neonatal hypoxic-ischemic encephalopathy in the future.
